# Performance of Fatty Liver Index in Identifying Non-Alcoholic Fatty Liver Disease in Population Studies. A Meta-Analysis

**DOI:** 10.3390/jcm10091877

**Published:** 2021-04-26

**Authors:** Marco Castellana, Rossella Donghia, Vito Guerra, Filippo Procino, Luisa Lampignano, Fabio Castellana, Roberta Zupo, Rodolfo Sardone, Giovanni De Pergola, Francesco Romanelli, Pierpaolo Trimboli, Gianluigi Giannelli

**Affiliations:** 1 Unit of Research Methodology and Data Sciences for Population Health “Salus in Apulia Study”, National Institute of Gastroenterology “Saverio de Bellis”, Research Hospital, Castellana Grotte, 70013 Bari, Italy; rossydonghia@gmail.com (R.D.); vito.guerra@irccsdebellis.it (V.G.); filippoprocino@yahoo.it (F.P.); luisa.lampignano@irccsdebellis.it (L.L.); castellanafabio@hotmail.it (F.C.); zuporoberta@gmail.com (R.Z.); rodolfo.sardone@irccsdebellis.it (R.S.); giovanni.depergola@uniba.it (G.D.P.); 2 Department of Biomedical Science and Human Oncology, University of Bari, 70121 Bari, Italy; 3 Department of Experimental Medicine, “Sapienza” University of Rome, 00185 Rome, Italy; francesco.romanelli@uniroma1.it; 4 Clinic of Endocrinology, Ente Ospedaliero Cantonale, 6900 Lugano, Switzerland; pierpaolo.trimboli@eoc.ch; 5 Faculty of Biomedical Sciences, Università della Svizzera Italiana (USI), 6900 Lugano, Switzerland; 6 National Institute of Gastroenterology “Saverio de Bellis”, Research Hospital, Castellana Grotte, 70013 Bari, Italy; gianluigi.giannelli@irccsdebellis.it

**Keywords:** fatty liver index, non-alcoholic fatty liver disease, steatosis, liver, meta-analysis

## Abstract

Background. Fatty liver index (FLI) is a non-invasive tool used to stratify the risk of non-alcoholic fatty liver disease (NAFLD) in population studies; whether it can be used to exclude or diagnose this disorder is unclear. We conducted a meta-analysis to assess the prevalence of NAFLD in each FLI class and the performance of FLI in detecting NAFLD. Methods. Four databases were searched until January 2021 (CRD42021231367). Original articles included were those reporting the performance of FLI and adopting ultrasound, computed tomography, or magnetic resonance as a reference standard. The numbers of subjects with NAFLD in FLI classes <30, 30–60, and ≥60, and the numbers of subjects classified as true/false positive/negative when adopting 30 and 60 as cut-offs were extracted. A random-effects model was used for pooling data. Results. Ten studies were included, evaluating 27,221 subjects without secondary causes of fatty liver disease. The prevalence of NAFLD in the three FLI classes was 14%, 42%, and 67%. Sensitivity, specificity, positive predictive value, negative predictive value, likelihood ratio for positive results, likelihood ratio for negative results, and diagnostic odds ratio were 81%, 65%, 53%, 84%, 2.3, 0.3, and 7.8 for the lower cut-off and 44%, 90%, 67%, 76%, 4.3, 0.6, and 7.3 for the higher cut-off, respectively. A similar performance was generally found in studies adopting ultrasound versus other imaging modalities. Conclusions. FLI showed an adequate performance in stratifying the risk of NAFLD. However, it showed only weak evidence of a discriminatory performance in excluding or diagnosing this disorder.

## 1. Introduction

Non-alcoholic fatty liver disease (NAFLD) is a common disorder with high prevalence, morbidity, and excess mortality rates, which has a major impact on affected subjects, their families, and the healthcare system. Globally, about one in four subjects are estimated to have this condition, and an even higher frequency is reported among specific populations [1,2]. In recent years, it has become the leading cause of chronic liver disease and the fastest-growing cause of liver transplantation [3,4]. The reference standard for the diagnosis of NAFLD is liver biopsy. However, it is common knowledge that this procedure can be considered only in a limited number of selected subjects owing to several issues. In fact, liver biopsy is invasive, costly, and can be associated with a small but not negligible risk of complications. Additionally, there is a discrepancy between the burden of NAFLD and the number of procedures that can be performed [5,6,7].

In order to overcome these limitations, non-invasive tools (NITs) have been introduced. The diagnosis of NAFLD relies on the detection of hepatic steatosis and the exclusion of secondary forms, including alcohol, viral infections, medications, and autoimmune and genetic disorders [5,6]. To detect hepatic steatosis, ultrasound, controlled-attenuation parameter measurement (CAP) by vibration-controlled transient elastography (VCTE), computed tomography (CT), or magnetic resonance (MR) modalities can be used. However, for larger-scale studies, serum biomarkers are preferred, as the availability and cost of imaging have a substantial impact on feasibility [6,8]. The best-validated tool is the fatty liver index (FLI), which is currently endorsed by both the European Association for the Study of the Liver, the European Association for the Study of Diabetes and the European Association for the Study of Obesity (EASL-EASD-EASO) guidelines, as well as the Asian Pacific Association for the Study of the Liver (APASL) guidelines [6,8]. FLI is a simple algorithm based on four commonly available parameters: waist circumference, body mass index, triglycerides, and gamma-glutamyl transferase (GGT). This tool was initially developed by Bedogni et al. to predict hepatic steatosis in the general population, and its reliability was later assessed in several studies [9,10,11]. In the original publication, FLI was presented as a tool to stratify the risk of hepatic steatosis, with scores below 30 being associated with low risk and 60 or higher with high risk [9].

Following the advent of this tool, several papers assessed its performance having NAFLD as target condition [12,13,14,15,16,17,18,19,20,21,22,23,24]. Of note, while FLI was used as a risk stratification tool in several studies, it was instead adopted as a diagnostic instrument in some of them. Specifically, in the latter studies, FLI scores below 30 were classified as non-NAFLD, between 30 and 60 as indeterminate, and 60 or higher as NAFLD [22,23,24]. The aim of the present study was to achieve solid information about the performance of FLI for these two purposes. Our research methodology envisaged a systematic search to identify population studies reporting data on imaging-diagnosed NAFLD and FLI. In addition, we performed a meta-analysis of available data to: (1) verify that FLI classes adequately stratify the risk of NAFLD; and (2) evaluate the sensitivity, specificity, positive predictive value (PPV), negative predictive value (NPV), likelihood ratio for positive results (LR+) and for negative results (LR−), and diagnostic odds ratio (DOR) of FLI < 30 in ruling out or ≥60 in ruling in NAFLD.

## 2. Materials and Methods

This meta-analysis was registered in PROSPERO (CRD42021231367) and performed in accordance with the PRISMA-DTA Statement (Tables S1 and S2) [25]. 

### 2.1. Search Strategy

A six-step search strategy was planned. Firstly, we searched for sentinel studies in PubMed. Secondly, we identified keywords in PubMed. Thirdly, the following complete search strategy was used in PubMed: (NAFLD[Title/Abstract]) AND (“fatty liver index” [Title/Abstract]). Fourthly, CENTRAL, Scopus, and Web of Science were searched using the same strategy. Fifthly, studies evaluating the performance of FLI in unselected subjects with imaging-diagnosed NAFLD were selected. Studies meeting the following criteria were excluded: (1) less than 100 subjects; (2) focusing on specific subgroups (e.g., pediatric, with or without type 2 diabetes, bariatric surgery subjects); (3) adopting CAP as a reference standard for diagnosis of NAFLD [26]; (4) adopting histology as a reference standard; (5) evaluating FLI other than the one developed by Bedogni et al. [9]; (6) letters, commentaries, and posters. Lastly, the references of included studies were searched to find additional papers. The last search was performed on 20 January 2021. No language restriction was adopted. Two investigators (M.C., F.P.) independently searched for papers, screened titles and abstracts of the retrieved articles, reviewed the full-texts, and selected articles for inclusion.

### 2.2. Data Extraction

The following information was extracted independently by the same investigators in a piloted form: (1) general information on the study (author, year of publication, country, study type, inclusion criteria, number of subjects); (2) cut-offs for the interpretation of FLI; (3) numbers of subjects with imaging-diagnosed NAFLD in each FLI class; (4) numbers of subjects classified as true/false positive/negative. Ultrasound, CT, or MR were the reference standards. FLI was the index test. FLI can be interpreted with a lower and an upper threshold, as stated (e.g., 30 and 60, respectively). Separate data extractions were performed, accordingly. A non-NAFLD subject was classified as true negative if the score was lower than the cut-off but false positive if the score was higher than the cut-off. In the same way, a NAFLD subject was classified as true positive if the score was higher than the cut-off but false negative if the score was lower than the cut-off. For each selected article, the main paper and supplementary data were searched; if data were missing, the authors were contacted via email. Data were cross-checked, and any discrepancy was discussed.

### 2.3. Study Quality Assessment

The risk of bias of included studies was assessed independently by two reviewers (M.C., F.P.), applying the Quality Assessment of Diagnostic Accuracy Studies (QUADAS-2) tool for the following aspects: patient selection; index test; reference standard; flow and timing. The risk of bias and concerns about applicability were rated as low, high, or unclear [27]. 

### 2.4. Data Analysis

The characteristics of included studies were summarized, and then separate analyses were performed according to the following steps. Firstly, a meta-analysis of proportion was carried to obtain the pooled rate with the 95% confidence interval (95%CI) of each FLI class among the evaluated subjects and of NAFLD within a specific FLI class. For statistical pooling of data, a random-effects model was used. Secondly, a meta-analysis of the diagnostic performance of FLI < 30 and ≥60 in excluding or selecting NAFLD was carried out. Summary operating points including sensitivity, specificity, NPV, PPV, LR+, LR-, and DOR, with the 95% CI, were estimated. DOR provides a single measure of test performance, equal to LR+/LR- and corresponding to the odds for a FLI score higher than the specific cut-off in a NAFLD subject compared with the odds for a FLI score higher than the specific cut-off in a non-NAFLD subject. Values ranged from zero to infinity, with higher values indicating higher performance. LR+ is the likelihood of obtaining a FLI score above the specific cut-off in a NAFLD subject (true positive) compared to the likelihood in a non-NAFLD subject (false positive). In the same way, a LR+ score higher than 10 indicates strong evidence, between 5 and 10 moderate evidence, and less than 5 weak evidence. LR- is the likelihood of obtaining a FLI score below the specific cut-off in a NAFLD subject (false negative) compared to the likelihood in a non-NAFLD subject (true negative). Again, a LR- less than 0.1 indicates strong evidence, between 0.1 and 0.2 moderate evidence, and higher than 0.2 weak evidence. A bivariate random-effects model was used for pooled analysis of the sensitivity and specificity; a random-effects model was used for pooled analysis of the remaining metrics [28]. A subgroup analysis according to the imaging modality for the diagnosis of NAFLD was conducted (e.g., ultrasound versus other imaging modalities). Heterogeneity between studies was assessed using I^2^, regarding 50% or higher values as high heterogeneity. For the proportion meta-analysis, funnel plots and Egger tests were carried out to evaluate the possible presence of significant publication bias. For the diagnostic performance meta-analysis, publication bias was not evaluated, due to uncertainty about the determinants for diagnostic accuracy studies and the inadequacy of tests for detecting funnel plot asymmetry [28]. All analyses were performed per subject using RevMan 5.4 (the Cochrane Collaboration, 2020, available online: https://training.cochrane.org/online-learning/core-software-cochrane-reviews/revman/revman-5-download, accessed on 1 February 2021) and STATA 16.0 (StataCorp software, 2019, Stata Statistical Software, Release 16, StataCorp LLC, College Station, TX, USA). Significance was set at *p* < 0.05.

## 3. Results

### 3.1. Study Characteristics

In total, 803 papers were found: 250 on PubMed, 49 on CENTRAL, 276 on Scopus, and 228 on Web of Science. One additional study was retrieved from a personal database [21]. After the removal of 488 duplicates, 316 articles were analyzed for title and abstract; 259 records were excluded (review, meta-analysis, commentary, conference papers, focusing on specific subgroups (e.g., pediatric, type 2 diabetes, bariatric surgery subjects, …), less than 100 patients, evaluating FLI other than the one developed by Bedogni et al. [9], adopting reference standards other than ultrasound, CT or MR, not within the field of the review). The remaining 57 papers were retrieved in full text, and 10 articles were finally included in the meta-analysis (Figure S1) [12,13,14,15,16,17,18,19,20,21]. No additional study was retrieved from references of included studies.

### 3.2. Qualitative Analysis 

The characteristics of the included articles are summarized in Table 1 [12,13,14,15,16,17,18,19,20,21]. The studies were published between 2013 and 2021 and had sample sizes ranging from 195 to 8626 patients. Five studies were cross-sectional, three prospective cohorts, and one retrospective cohort; the design was not reported in one study [18]. One study was conducted in Brazil, one in China, one in Israel, one in Italy, one in Japan, one in Korea, one in Spain, one in Taiwan, one in the Netherlands, and one in the United States of America. Participants were generally adult subjects without secondary causes of fatty liver disease (FLD); pregnant women were excluded in two studies [15,16,17,18] and subjects with known liver disease (e.g., cirrhosis) in four [14,15,17,19]. NAFLD was diagnosed by ultrasound in seven studies; Jung et al. and McHenry et al. adopted MR as a reference standard, while Carvalho Goulart et al. employed CT [15,19,20]. The prevalence of NAFLD ranged from 26% in Arteaga et al. to 46% in Chen et al. [14,18]. The performance of both the lower and the higher cut-offs of FLI was generally evaluated, the only exception being Zelber-Sagi et al., who assessed only the higher cut-off [13]. Overall, 27,221 subjects were included; 8273 were diagnosed with NAFLD.

### 3.3. Quantitative Analysis 

**Figure 1:**
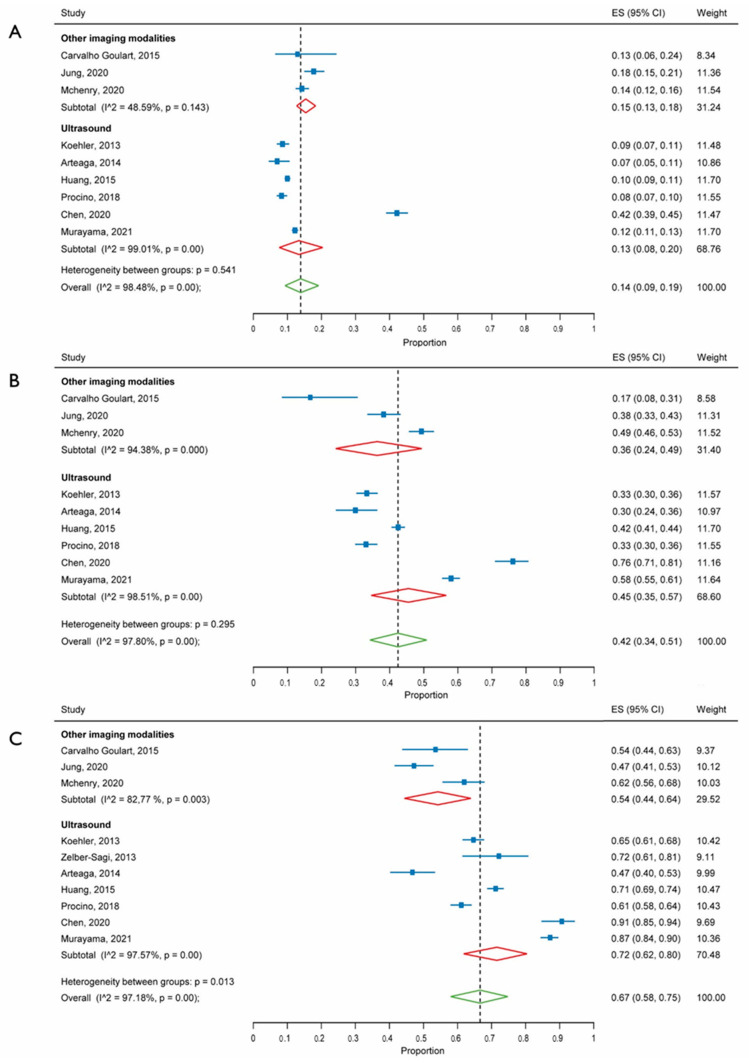
Forest plot of the prevalence of non-alcoholic fatty liver disease in subjects with fatty liver index below 30 (**A**), between 30 and 60 (**B**), or 60 or higher (**C**). 95% CI, 95% confidence interval. References: Koehler, 2013 [12], Zelber-Sagi, 2013 [13], Arteaga, 2014 [14], Carvalho Goulart, 2015 [15], Huang, 2015 [16], Procino, 2018 [17], Chen, 2020 [18], Jung, 2020 [19], McHenry, 2020 [20], Murayama, 2021 [21].

First, the pooled prevalence of each FLI class among the included subjects and of NAFLD in each FLI class was assessed. The overall prevalence was 49% (95% CI: 40 to 58) for the FLI < 30 class, 27% (95% CI: 23 to 30) for the FLI 30–60 class, and 23% (95% CI: 18 to 29) for the FLI ≥ 60 class. The pooled prevalence of NAFLD was 14% (95%CI: 9 to 19) in the FLI < 30 class, 42% (95% CI: 34 to 51) in the FLI 30–60 class, and 67% (95% CI: 58 to 75) in the FLI ≥ 60 class. There was no difference according to the imaging modality for the diagnosis of NAFLD in the first two FLI classes; whereas in the highest class, a higher prevalence of NAFLD was estimated in studies adopting ultrasound versus CT/MR as a reference standard (72% versus 54%; *p* = 0.01). High heterogeneity was found for all the outcomes (Figure 1). There was no evidence of publication bias (Figure S2).

**Figure 2:**
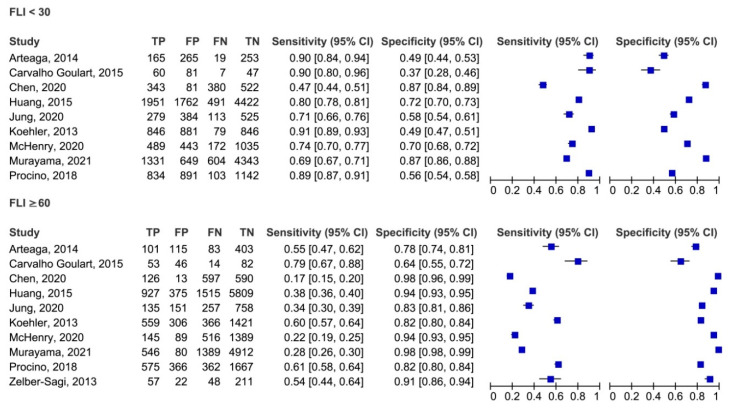
Forest plot of the sensitivity and specificity of fatty liver index in identifying non-alcoholic fatty liver disease according to the lower and the higher cut-off. FLI, fatty liver index; FN, false negative; FP, false positive; TN, true negative; TP, true positive; 95% CI, 95% confidence interval. References: Koehler, 2013 [12], Zelber-Sagi, 2013 [13], Arteaga, 2014 [14], Carvalho Goulart, 2015 [15], Huang, 2015 [16], Procino, 2018 [17], Chen, 2020 [18], Jung, 2020 [19], McHenry, 2020 [20], Murayama, 2021 [21].

Second, a diagnostic performance meta-analysis of FLI < 30 or ≥60 in excluding or identifying NAFLD was carried out. Forest plots of the sensitivity and specificity of FLI interpreted according to the lower or the higher cut-off are shown in Figure 2. For the lower cut-off, the pooled sensitivity was 81%, specificity was 65%, PPV was 53%, and NPV was 84%. For the higher cut-off, the pooled sensitivity was 44%, specificity was 90%, PPV was 67%, and NPV was 76%. Because these summary operating points are influenced by the prevalence of the disease in the population tested, we estimated the following parameters, which are independent of disease prevalence and thus characteristics of FLI. The pooled LR+ were 2.3 and 4.3, LR- were 0.3 and 0.6, and DOR were 7.8 and 7.3, respectively. A similar performance was found when the reference standard for the diagnosis of NAFLD was assessed (Table S3). High heterogeneity was found for all the outcomes (Table 2).

### 3.4. Study Quality Assessment 

The risk of bias of the included studies is shown in Table S4. Overall, a consecutive or random sample of subjects was included who underwent ultrasound/CT/MR and had a final diagnosis of NAFLD during a specific period; FLI was calculated according to objective parameters (e.g., body mass index, GGT, triglycerides, waist circumference) and interpreted according to standard cut-offs (e.g., 30 and 60). Concerning reference standard bias, liver biopsy is the gold standard for the diagnosis and staging of NAFLD. The performance of imaging modalities in diagnosing steatosis is significant but suboptimal, therefore the corresponding item for the risk of bias was rated as high [29]. Additionally, eight studies diagnosed NAFLD after excluding some but not all the main secondary causes of FLD, therefore the applicability concerns for the reference standard were rated as high [12,14,15,16,17,19,20,21]. Patient selection applicability concerns for Koehler et al. were rated as high because only subjects aged 55 or older were included [12]. Finally, several studies did not report data allowing the assessment of the patient selection nor the flow and timing risks of bias [13,15,16,17,18,19,20,21].

## 4. Discussion

The aim of this meta-analysis was to identify the best available evidence on the performance of FLI in stratifying the risk of NAFLD and ruling in or ruling out this condition in large samples of unselected subjects. An extensive database search was performed without time or language restrictions, and inclusion criteria were defined prior to the database search. To our knowledge, this is the first meta-analysis of the topic; it was based on independent summary operating measures, allowing studies evaluating populations with a different prevalence of NAFLD to be interpreted together.

Ten studies were found, evaluating the performance of FLI among 8273 subjects diagnosed with and 18,948 subjects without NAFLD. Of note, these studies generally excluded only those subjects with pregnancy, known liver disease, or secondary causes of FLD. No study selected subjects according to their comorbidities (e.g., with or without type 2 diabetes) or anthropometric data (e.g., with or without obesity). Indeed, even if an age criterion for eligibility was reported in all of them, it resulted in the enrollment of an elderly portion of the population only in Koehler et al. [12]. Additionally, the overall prevalence of NAFLD was close to the figure estimated globally in a recent meta-analysis [1]. This is the basis for considering the included studies to be affected by a low selection bias and our results to be potentially applicable to populations other than those reported in the analyses.

The prevalence of NAFLD was 14% in the FLI class below 30, 42% in the FLI class between 30 and 60, and 67% in the FLI class scoring 60 or higher. On the one hand, these findings support the use of FLI as a tool to stratify the risk of NAFLD in population studies. On the other hand, they seem to discourage the use of FLI to diagnose or exclude NAFLD. In order to gain more insight into the latter application, a diagnostic performance meta-analysis was conducted. Indeed, FLI, like other NITs, was conceived to distinguish using commonly available anthropometric and laboratory data subjects at low risk from those at high risk of NAFLD according to a score below the lower cut-off or higher than the higher cut-off. The risk of NAFLD cannot be adequately stratified in those subjects scoring between the lower and the higher cut-offs (i.e., indeterminate); other strategies need to be considered therefore in these subjects only (e.g., in a population study reviewing data on ultrasound) [9]. The present meta-analysis challenges the diagnostic use of FLI. First, when the lower cut-off was considered, a sensitivity of 81%, NPV of 84%, and LR- of 0.3 were found, providing only weak evidence of a discriminatory performance. Second, when the higher cut-off was considered, a specificity of 90%, PPV of 67%, and LR+ of 4.3 were found, again providing only weak evidence of a discriminatory performance. Third, when the dual-threshold strategy was adopted, about one in four patients were classified as indeterminate, corresponding to the prevalence of subjects with a FLI score between 30 and 60. Applying the results of our analyses to a hypothetical population, some considerations may be drawn. Specifically, if subjects with a score below the lower cut-off were diagnosed as non-NAFLD, about one in six patients would have been incorrectly classified. In the same way, if subjects with a score higher than the higher cut-off were considered as affected by NAFLD, imaging would have confirmed this diagnosis only in two in three patients. Finally, if the records of only subjects with a score between the lower and the higher cut-off were reviewed for an imaging-based diagnosis of NAFLD, the number of data checks would have been reduced by 73%, but the limitations of the single strategies would still apply. In short, our data do not support the view of FLI as a reliable tool to diagnose or exclude NAFLD. Rather, it should be considered as a tool serving to stratify the risk of NAFLD but with only a weak diagnostic performance, highlighting the need for better markers. Until these tools are developed and validated, we should continue to rely on imaging. Specifically, in a population study perspective, ultrasound should still be considered as a reference standard, being commonly available, safe, low cost and having no contraindications.

Limitations of the present paper should be discussed. Firstly, the aim of the present meta-analysis was to assess the performance of FLI for NAFLD in population studies. However, in current guidelines, the use of FLI is recommended to diagnose steatosis [6,8]. In the original publication, patients with HBV or HCV infection were excluded, as were subjects with other secondary forms of FLD in the studies included in the present paper [9]. Therefore, the results of the present meta-analysis are reliable from a NAFLD perspective, as there are no differences between excluding secondary forms of FLD before or after an imaging-, blood-biomarker-, blood-score-, or histology-based diagnosis of steatosis. They are not applicable to the recently proposed definition of metabolic associated fatty liver disease (MAFLD), according to which the presence of just overweight/obesity, type 2 diabetes, or risk factors allows the classification of a subject with FLD as either MAFLD or non-MAFLD [6,8]. Secondly, the exclusion of studies adopting liver biopsy as a reference standard resulted in a high risk of bias for the diagnosis of NAFLD. Nevertheless, this was planned to reduce the selection bias to smaller studies with more severe forms. One study with an adequate sample size was conducted by Fedchuck et al.: 324 patients with clinical or ultrasound suspicion of NAFLD and who underwent liver biopsy were included; the prevalence of steatosis and advanced fibrosis was 95% and 24%, respectively. A FLI score of 60 or higher was associated with a sensitivity of 76%, specificity of 87%, PPV of 99%, and NPV of 15% [30]. These estimates correspond to a LR+ of 5.7, LR- of 0.3, and DOR of 20.7. Therefore, our findings of limited evidence of a discriminatory performance of FLI are confirmed even when a population is affected by a high selection bias, but a low risk of bias for the reference standard is considered. Thirdly, the included studies were affected by a variable bias concerning the clinical diagnosis of NAFLD. While alcohol and viral infection were excluded as secondary forms of FLD in most of the studies, other known causes including medications, autoimmune, genetic, or metabolic disorders were evaluated only in some of them. Nevertheless, it is common knowledge that the prevalence of these secondary forms of FLD is low in the general population [31,32]. Lastly, our results were characterized by high heterogeneity. Several factors may explain this finding: (1) differences in the included subjects (e.g., ethnicity); (2) clinical factors considered for the diagnosis of NAFLD in subjects with FLD, as stated; and (3) imaging modalities for the diagnosis of NAFLD, as ultrasound is operator dependent [33]. This indirectly supports the poor performance of FLI as a diagnostic tool. On the other hand, given the absolute values of the prevalence of NAFLD in each FLI class, its ability to stratify the risk of NAFLD would be little affected.

## 5. Conclusions

The high prevalence and clinical relevance of NAFLD have prompted the scientific community to develop non-invasive tools with the aim of assessing the individual risk of steatosis or fibrosis and to facilitate the conduction of large studies. FLI is a practical instrument, based on commonly available data, and is the only non-invasive tool currently recommended for the assessment of steatosis. In the present study, only studies with a low selection bias were included and FLI was found to be effective in stratifying the risk of NAFLD. About one in six subjects classified as FLI < 30 were confirmed to be affected by NAFLD, compared to about two in three in those of those classified as FLI ≥ 60. Conversely, only a weak performance was found when assessing its potential application to exclude or diagnose NAFLD. Further prospective studies would be helpful to further support the performance of the FLI and assess its role in the diagnosis of MAFLD.

## Figures and Tables

**Table 1:** Characteristics of included studies and availability of data.

First Author, Year	Country	Study Design	Number of Patients	Population	Reference Standard	NAFLD (%)	FLI < 30	FLI ≥ 60
Koehler, 2013 [12]	The Netherlands	PCS	2652	≥55 years, without secondary causes of FLD (alcohol, virus, drugs)	US	925 (35%)	x	x
Zelber-Sagi, 2013 [13]	Israel	Cross-sectional	338	24–70 years, without secondary causes of FLD (alcohol, virus, drugs, inflammatory bowel disease, prior surgery that could cause FLD, or celiac disease)	US	105 (31%)	-	x
Arteaga, 2014 [14]	Spain	Cross-sectional	702	15–85 years, without known liver diseases or secondary causes of FLD (alcohol, virus)	US	184 (26%)	x	x
Carvalho Goulart, 2015 [15]	Brazil	Cross-sectional	195	35–75 years, without pregnancy, known liver disease or secondary causes of FLD (alcohol, virus)	CT	67 (34%)	x	x
Huang, 2015 [16]	China	Cross-sectional	8626	≥40 years, without secondary causes of FLD (alcohol, virus, drugs, autoimmune disorders)	US	2442 (28%)	x	x
Procino, 2018 [17]	Italy	PCS	2970	≥30 years, without known liver disease or secondary causes of FLD (alcohol, virus, autoimmune or genetic disorders)	US	937 (32%)	x	x
Chen, 2020 [18]	Taiwan	-	1371	>30 years, without pregnancy or secondary causes of FLD (alcohol, virus, drugs, gastric bypass surgery, autoimmune, genetic or metabolic disorders)	US	625 (46%)	x	x
Jung, 2020 [19]	Korea	RCS	1301	>30 years, without known liver diseases or secondary causes of FLD (alcohol, virus, autoimmune, genetic or metabolic disorders)	MR	392 (30%)	x	x
McHenry, 2020 [20]	USA	PCS	2139	18–65 years, without secondary causes of FLD (alcohol)	MR	661 (31%)	x	x
Murayama, 2021 [21]	Japan	Cross-sectional	6927	21–86 years, without secondary causes of FLD (alcohol, virus)	US	1935 (28%)	x	x

CT, computed tomography; FLD, fatty liver disease; FLI, fatty liver index; MR, magnetic resonance; NAFLD, non-alcoholic fatty liver disease; PCS, prospective cohort study; RCS, retrospective cohort study; US, ultrasound; -, not reported; x, retrieved data.

**Table 2:** Summary estimates of the fatty liver index in identifying non-alcoholic fatty liver disease according to the lower and the higher cut-off.

Cut-Off	Number of Subjects (Number of Studies)	Prevalence of Non-Alcoholic Fatty Liver Disease (95% CI)	Sensitivity (95% CI)	Specificity (95% CI)	Positive Predictive Value (95% CI)	Negative Predictive Value (95% CI)	Likelihood Ratio for Positive Results (95% CI)	Likelihood Ratio for Negative Results (95% CI)	Diagnostic Odds Ratio (95% CI)
<30	26,838 (9)	32 (29 to 35)	81 (71 to 88)	65 (52 to 76)	53 (45 to 61)	84 (80 to 89)	2.32 (1.82 to 2.95)	0.30 (0.24 to 0.38)	7.83 (5.80 to 10.57)
≥60	27,176 (10)	32 (29 to 35)	44 (33 to 55)	90 (84 to 94)	67 (57 to 74)	76 (72 to 81)	4.29 (30.4 to 6.05)	0.59 (0.50 to 0.69)	7.25 (5.03 to 10.45)

95% CI, 95% confidence interval.

## Data Availability

No new data were created or analyzed in this study. Data sharing is not applicable to this article.

## Supplementary Materials

The following are available online at https://www.mdpi.com/article/10.3390/jcm10091877/s1, Figure S1: Flow-chart of the systematic review, Figure S2: Publication bias of the prevalence of NAFLD in each FLI class, Table S1: PRISMA-DTA for Abstract Checklist, Table S2: PRISMA-DTA Checklist, Table S3: Summary estimates of the FLI in identifying NAFLD according to the lower and the higher cut-off, and imaging modality for the diagnosis of NAFLD, Table S4: Summary of risk of bias and applicability concerns: review authors’ judgments about each domain for each study included.
