# Supplementary material for: Performance of Fatty Liver Index in Identifying Non-Alcoholic Fatty Liver Disease in Population Studies. A Meta-Analysis
Source: J Clin Med. 2021 Apr 26;10(9):1877. doi: 10.3390/jcm10091877 (PMC8123596; doi:10.3390/jcm10091877)

Figure S2: Publication bias of the prevalence of non-alcoholic fatty liver disease in each fatty liver index class.

Fatty liver index score below 30

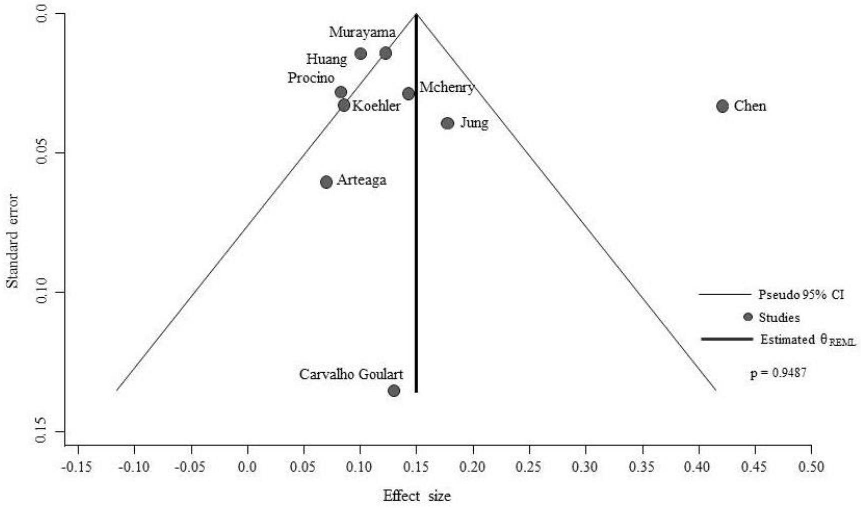

Fatty liver index score between 30 and 60

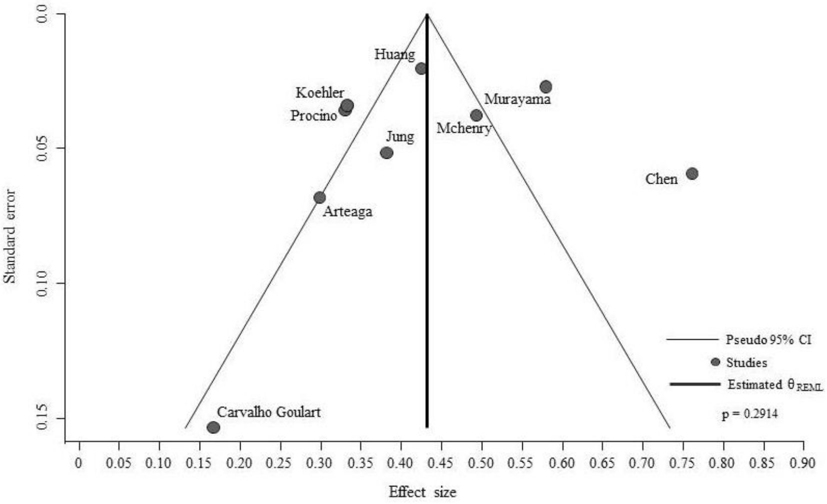

Fatty liver index score of 60 or higher

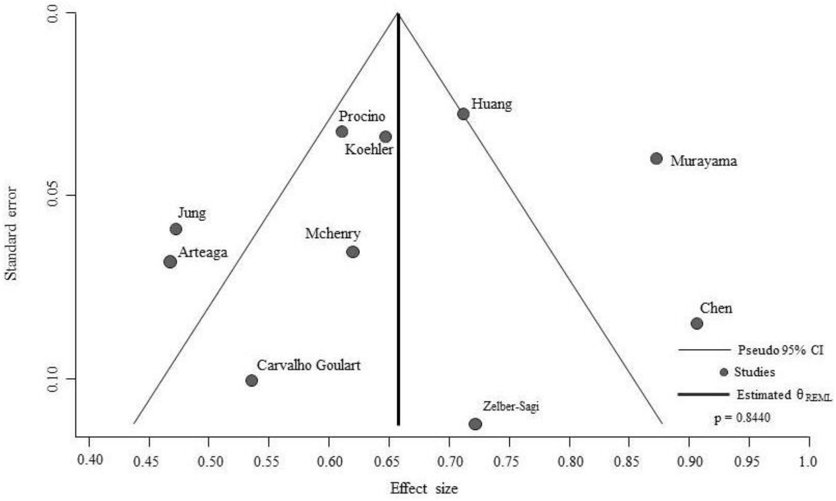

Supplement: Supplementary file 1 [file jcm-10-01877-s001.zip › jcm-1185782-supply/Figure S2_R1.pdf]
