# Supplementary material for: Performance of Fatty Liver Index in Identifying Non-Alcoholic Fatty Liver Disease in Population Studies. A Meta-Analysis
Source: J Clin Med. 2021 Apr 26;10(9):1877. doi: 10.3390/jcm10091877 (PMC8123596; doi:10.3390/jcm10091877)
Supplement: Supplementary file 1 [file jcm-10-01877-s001.zip › jcm-1185782-supply/Table S1.pdf]

Table S1: PRISMA-DTA for Abstract Checklist.

Section/topic	#	PRISMA-DTA for Abstracts Checklist item	Reported on page #
**TITLE and PURPOSE**			
Title	1	Identify the report as a systematic review (+/- meta-analysis) of diagnostic test accuracy (DTA) studies.	3
Objectives	2	Indicate the research question, including components such as participants, index test, and target conditions.	3
**METHODS**			
Eligibility criteria	3	Include study characteristics used as criteria for eligibility.	3
Information sources	4	List the key databases searched and the search dates.	3
Risk of bias & applicability	5	Indicate the methods of assessing risk of bias and applicability.	-
Synthesis of results	A1	Indicate the methods for the data synthesis.	3
**RESULTS**			
Included studies	6	Indicate the number and type of included studies and the participants and relevant characteristics of the studies (including the reference standard).	3
Synthesis of results	7	Include the results for the analysis of diagnostic accuracy, preferably indicating the number of studies and participants. Describe test accuracy including variability; if meta-analysis was done, include summary results and confidence intervals.	3
**DISCUSSION**			
Strengths and limitations	9	Provide a brief summary of the strengths and limitations of the evidence	-
Interpretation	10	Provide a general interpretation of the results and the important implications.	3
**OTHER**			
Funding	11	Indicate the primary source of funding for the review.	2
Registration	12	Provide the registration number and the registry name	3

Adapted From: McInnes MDF, Moher D, Thombs BD, McGrath TA, Bossuyt PM, The PRISMA-DTA Group (2018). Preferred Reporting Items for a Systematic Review and Meta-analysis of Diagnostic Test Accuracy Studies: The PRISMA-DTA Statement. JAMA. 2018 Jan 23;319(4):388-396. doi: 10.1001/jama.2017.19163.

For more information, visit: **www.prisma-statement.org**.

Page 1 of 1
