# Supplementary material for: Performance of Fatty Liver Index in Identifying Non-Alcoholic Fatty Liver Disease in Population Studies. A Meta-Analysis
Source: J Clin Med. 2021 Apr 26;10(9):1877. doi: 10.3390/jcm10091877 (PMC8123596; doi:10.3390/jcm10091877)
Supplement: Supplementary file 1 [file jcm-10-01877-s001.zip › jcm-1185782-supply/Table S3_R1.pdf]

Table S3: Summary estimates of the fatty liver index in identifying non-alcoholic fatty liver disease according to the lower and the higher cut-off, and imaging modality for the diagnosis of NAFLD.

Cut-off	Number of subjects (number of studies)	Prevalence of non-alcoholic fatty liver disease (95% CI)	Sensitivity (95% CI)	Specificity (95% CI)	Positive predictive value (95%CI)	Negative predictive value (95%CI)	Likelihood ratio for positive results (95% CI)	Likelihood ratio for negative results (95% CI)	Diagnostic odds ratio (95% CI)
<30	26,838 (9)	32 (29 to 35)	81 (71 to 88)	65 (52 to 76)	53 (45 to 61)	84 (80 to 89)	2.32 (1.82 to 2.95)	0.30 (0.24 to 0.38)	7.83 (5.80 to 10.57)
Ultrasound	23,203 (6)	33 (28 to 37)	81 (69 to 89)	69 (54 to 81)	56 (47 to 65)	85 (80 to 81)	2.62 (1.93 to 3.68)	0.27 (0.21 to 0.35)	9.8 (7.6 to 12.5)
Other imaging modalities	3,635 (3)	31 (29 to 32)	76 (69 to 82)	56 (42 to 69)	46 (38 to 54)	84 (82 to 87)	1.81 (1.31 to 2.50)	0.38 (0.28 to 0.50)	4.8 (2.8 to 8.3)
≥60	27,176 (10)	32 (29 to 35)	44 (33 to 55)	90 (84 to 94)	66 (57 to 74)	76 (72 to 81)	4.3 (3.4 to 6.05)	0.59 (0.50 to 0.69)	7.25 (5.03 to 10.45)
Ultrasound	23,541 (7)	32 (29 to 36)	44 (32 to 57)	92 (85 to 96)	71 (61 to 80)	76 (71 to 81)	5.38 (3.56 to 8.13)	0.58 (0.49 to 0.67)	9.14 (6.39 to 13.06)
Other imaging modalities	3,635 (3)	31 (29 to 32)	44 (23 to 66)	84 (64 to 94)	54 (44 to 64)	76 (72 to 81)	2.54 (1.79 to 3.60)	0.60 (0.34 to 1.05)	3.97 (2.47 to 6.39)

95% CI, 95% confidence interval
