# Supplementary material for: Performance of Fatty Liver Index in Identifying Non-Alcoholic Fatty Liver Disease in Population Studies. A Meta-Analysis
Source: J Clin Med. 2021 Apr 26;10(9):1877. doi: 10.3390/jcm10091877 (PMC8123596; doi:10.3390/jcm10091877)
Supplement: Supplementary file 1 [file jcm-10-01877-s001.zip › jcm-1185782-supply/Table S4.pdf]

Table S4: Summary of risk of bias and applicability concerns: review authors' judgments about each domain for each study included.

	Risk of bias				Applicability concerns		
**First Author, year**	**Patient selection**	**Index test**	**Reference standard**	**Flow and timing**	**Patient selection**	**Index test**	**Reference standard**
Koehler, 2013 [12]	Low	Low	High	Low	High	Low	High
Zelber-Sagi, 2013 [13]	Low	Low	High	Unclear	Low	Low	Low
Arteaga, 2014 [14]	Low	Low	High	Low	Low	Low	High
Carvalho Goulart, 2015 [15]	Unclear	Low	High	Low	Low	Low	High
Huang, 2015 [16]	Unclear	Low	High	Unclear	Low	Low	High
Procino, 2018 [17]	Low	Low	High	Unclear	Low	Low	High
Chen, 2020 [18]	Unclear	Low	High	Unclear	Low	Low	Low
Jung, 2020 [19]	Unclear	Low	High	Unclear	Low	Low	High
McHenry, 2020 [20]	Low	Low	High	Unclear	Low	Low	High
Murayama, 2021 [21]	Unclear	Low	High	Unclear	Low	Low	High
