# Supplementary figures and images for: Performance of Fatty Liver Index in Identifying Non-Alcoholic Fatty Liver Disease in Population Studies. A Meta-Analysis
Source: J Clin Med. 2021 Apr 26;10(9):1877. doi: 10.3390/jcm10091877 (PMC8123596; doi:10.3390/jcm10091877)

**Figure S1: Flow-chart of the systematic review.**

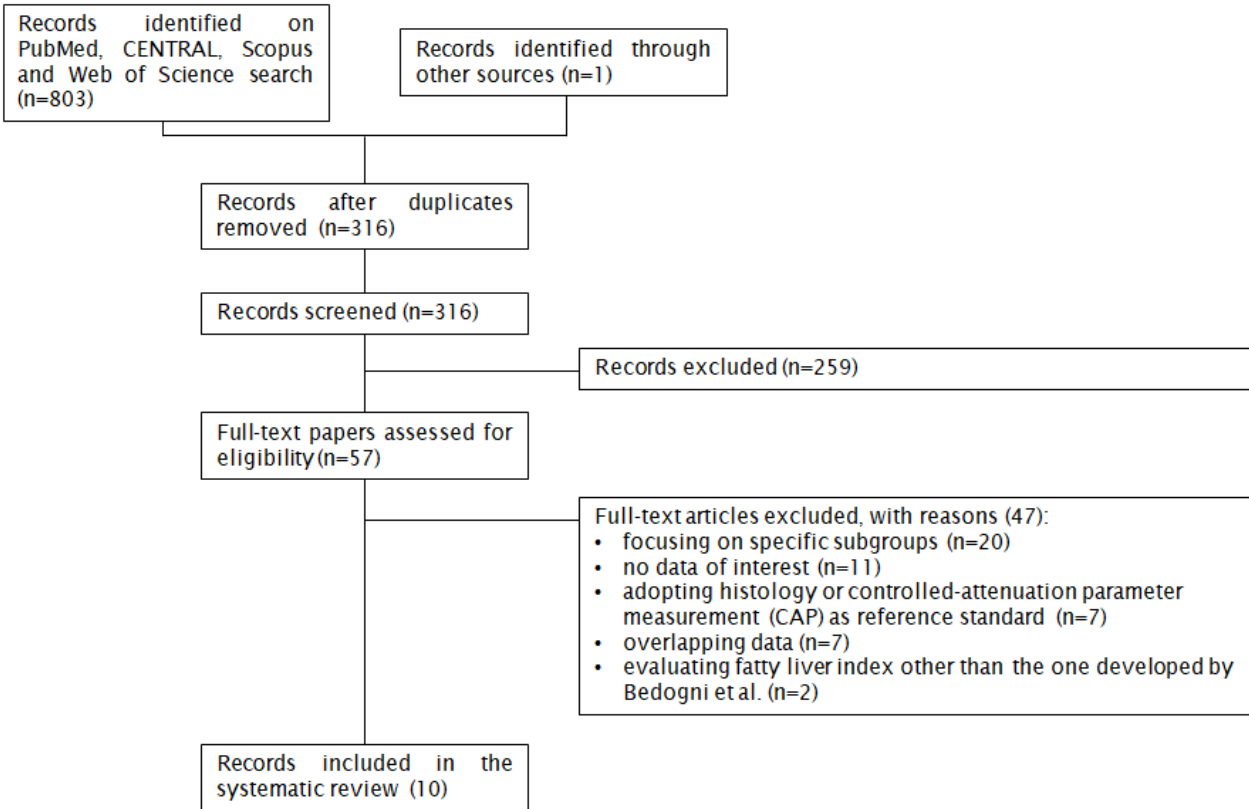

Supplement: Supplementary file 1 [file jcm-10-01877-s001.zip › jcm-1185782-supply/Figure S1_R1.pdf]
